# Habitats as Complex Odour Environments: How Does Plant Diversity Affect Herbivore and Parasitoid Orientation?

**DOI:** 10.1371/journal.pone.0085152

**Published:** 2014-01-09

**Authors:** Nicole Wäschke, Kristin Hardge, Christine Hancock, Monika Hilker, Elisabeth Obermaier, Torsten Meiners

**Affiliations:** 1 Freie Universität Berlin, Institute of Biology, Applied Zoology / Animal Ecology, Berlin, Germany; 2 University of Würzburg, Department of Animal Ecology and Tropical Biology, Würzburg, Germany; Rutgers University, United States of America

## Abstract

Plant diversity is known to affect success of host location by pest insects, but its effect on olfactory orientation of non-pest insect species has hardly been addressed. First, we tested in laboratory experiments the hypothesis that non-host plants, which increase odour complexity in habitats, affect the host location ability of herbivores and parasitoids. Furthermore, we recorded field data of plant diversity in addition to herbivore and parasitoid abundance at 77 grassland sites in three different regions in Germany in order to elucidate whether our laboratory results reflect the field situation. As a model system we used the herb *Plantago lanceolata*, the herbivorous weevil *Mecinus pascuorum*, and its larval parasitoid *Mesopolobus incultus.* The laboratory bioassays revealed that both the herbivorous weevil and its larval parasitoid can locate their host plant and host *via* olfactory cues even in the presence of non-host odour. In a newly established two-circle olfactometer, the weeviĺs capability to detect host plant odour was not affected by odours from non-host plants. However, addition of non-host plant odours to host plant odour enhanced the weeviĺs foraging activity. The parasitoid was attracted by a combination of host plant and host volatiles in both the absence and presence of non-host plant volatiles in a Y-tube olfactometer. In dual choice tests the parasitoid preferred the blend of host plant and host volatiles over its combination with non-host plant volatiles. In the field, no indication was found that high plant diversity disturbs host (plant) location by the weevil and its parasitoid. In contrast, plant diversity was positively correlated with weevil abundance, whereas parasitoid abundance was independent of plant diversity. Therefore, we conclude that weevils and parasitoids showed the sensory capacity to successfully cope with complex vegetation odours when searching for hosts.

## Introduction

Host location is a crucial event in an insect’s life. It is a prerequisite for accessing food or oviposition sites (reviewed by [1], [2]). Herbivores as well as parasitoids use volatile cues of the host plant, the host, or the microhabitat for locating their hosts at greater distances (reviewed by [3], [4]). However, multitrophic interactions take place in heterogeneous and complex environments, formed primarily by both host and non-host plants [5].

Plant diversity is known to affect host location behaviour of herbivores [6]–[8] and carnivores [9]–[11]. The plant species composition of a community may determine the detectability of food plants for herbivores as well as the detectability of host insects for parasitoids [12]–[14]. The plethora of physical structures in complex and diverse vegetation may affect insect host foraging behaviour [15]. Furthermore, vegetation odour can significantly impact upon olfactory orientation of insects. Non-host plants and high plant diversity may form a complex odour bouquet which insects have to cope with when foraging for their hosts [12], [16]–[18].

Results of laboratory studies on insect olfactory orientation to host volatiles often differ from insect olfactory behaviour observed in field studies [12]. Many laboratory studies neglect the impact of the complex odour bouquets present in the natural habitat [19]. Thus, combined field and laboratory studies are necessary in order to elucidate the impact of non-host plants on the orientation of herbivores and their natural enemies.

Laboratory studies have revealed that the effects of diverse odorous surroundings of a host plant or host may be manifold, e.g. positive for herbivores and their parasitoids, negative for both or for just one trophic level (reviewed by [8], [20]). Non-host plant odours can mask the target odour [12], [21], [22] or may have a repellent effect [23], [24]. However, some insects are not disturbed by the diversity of odours released from other environmental sources present in the habitat where they are searching for a host [25], [26]. Background (habitat) odour may indicate the presence of a host and even lead to the increased attraction of insects [27], [28].

Thus far, research on the effects of plant diversity on insects has focused primarily on crop plants and the orientation behaviour of insect pest species [12], [16]. However, agricultural systems do not function like natural ecosystems where members of a food web may adapt to each other in the course of evolution. Insects living in natural habitats might respond differently to environmental factors than those in agricultural ecosystems [3], [29]. So far, only a few studies have focused on odour-mediated interactions between non-crop plant species and members of higher trophic levels ([30]; and see e.g. [31]–[34]).

In the present study we combine a laboratory and a field approach to examine the impact of plant (odour) diversity on host location in a tritrophic system by using the perennial herb *Plantago lanceolata* L. (Plantaginaceae), the herbivorous weevil *Mecinus pascuorum* (Gyllenhal) (Coleoptera: Curculionidae), and its larval parasitoid *Mesopolobus incultus* (Walker) (Hymenoptera: Pteromalidae) as a model system. In order to mimic natural odorous conditions in the lab we established a new olfactometer assay and tested (1) whether weevil adults are attracted by odour of their host plant, and if so, whether this attraction is affected by plant diversity (presence of non-host plants). Furthermore, we investigated (2) whether the parasitoid is attracted to odour of the “host complex” consisting of the host plant and the host insect, and how plant diversity affects the parasitoid olfactory orientation to the host complex. In the field we studied (3) the impact of plant diversity on the abundance of the herbivorous weevil and its parasitoid; we surveyed vegetation data (number of plant species and their abundances) and abundances of the weevils and their parasitoids at grassland plots differing in plant diversity within a large scale project in Germany [35]. Abundances of herbivores and parasitoids were determined since they provide information on how successfully a habitat may be colonised, i.e. they are indicators of host resource availability and foraging efficiency for a host (plant).

## Materials and Methods

### Ethics statement

Field work permits were issued by the responsible state environmental offices of Baden-Württemberg, Thuringia, and Brandenburg (according to §72 BbgNatSchG).

### Study system

The ubiquitous herb *P. lanceolata* is native to Europe [36]. It occurs on meadows and pastures and is widespread in habitats with different plant diversities. The specialised weevil *M. pascuorum* oviposits in the seeds of *P. lanceolata* in June and July. Weevil larvae develop within the seeds of *P. lanceolata* inflorescences and emerge from August to September. These larvae are hosts of the generalist parasitic wasp *M. incultus* which attacks larvae feeding inside the seeds of *P. lanceolata* inflorescences [37] as well as other coleopteran insect larvae feeding on *Plantago* and *Trifolium* plants [38].

### Laboratory assays

**Effect of plant diversity on olfactory orientation of the herbivore.** *Insects and plants* **.** Weevils used for the laboratory olfactometer bioassays were collected from June to July 2009 when female weevils were searching for oviposition sites [37], [39]. To reduce a possible impact of sampling in the studied regions, the weevils were not collected at the Biodiversity Exploratory plots (for details see below “Field study”), but at a site called Wuhletal (Berlin Marzahn-Hellersdorf, Germany). The weevils were reared at 23°C ± 1°C, 48% rh and 14:10 LD. Since weevils were collected in the field, intrinsic factors that may affect oviposition, i.e. age, egg load, mating status or oviposition experience, were unknown. In the laboratory, male and female weevils were kept together for at least one week to ensure mating before separating the sexes. Weevils were fed daily with fresh host plant material.

All plants that were used for the laboratory assays with weevils were grown in a greenhouse at 24°C to 30°C, 20% to 34% rh, and 14:10 LD. They were grown from seeds that were obtained from Botanical Garden Berlin and sown in soil (Einheitserde Typ T Topferde, Einheitserde- und Humuswerke Gebr. Patzer GmbH & Co. KG, Sinntal - Jossa, Germany). Plants were grown individually in pots (6 cm×6 cm×8 cm) after four to five weeks. At the same time, pots were filled with soil and were later used as control. Seven- to nine-week-old plants were used in the bioassays; when plants of this age were used, they still fit into the two-circle olfactometer set-up described below; furthermore, the *P. lanceolata* plants were flowering at this age and thus, have reached a stage at which they display their inflorescences, i.e. the oviposition sites for the weevils.

The two herbs *Achillea millefolium* L. (Asteraceae) and *Agrimonia eupatoria* L. (Rosaceae) (tested when they had developed 11 and 12 fully expanded leaves, respectively) and the grasses *Festuca rubra* L. and *Poa pratensis* L. (both Poaceae) (tested when they had developed 24 and 16 leaves, respectively) occur in the natural habitat of the weevils and co-occur with the host plant *P. lanceolata*. These non-host plant species were used here in order to generate a complex odour blend that mimics the natural habitat odour. The non-host plant species occurred in both the flowering and non-flowering stage in the field during the weeviĺs oviposition period and flowering time of *P. lanceolata*; the flowering time of the non-host plants growing in the different field plots varied due to the different environmental factors the plots were exposed to. In order to ensure consistent conditions for the laboratory bioassays, we decided to use all non-host plants in a non-flowering stage.

### General olfactometer setup

**Figure 1:**
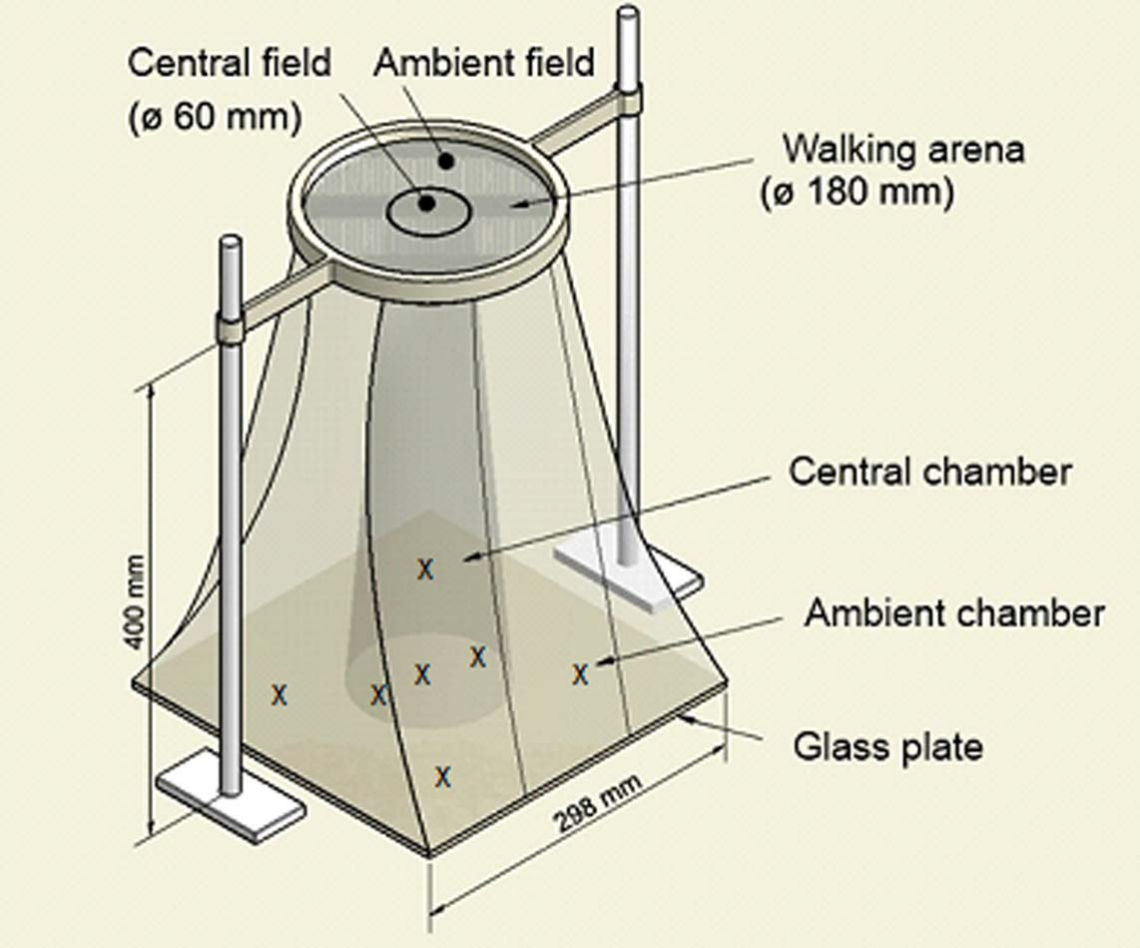
Two-circle olfactometer. The diameter of the whole arena is 180(ø 60 mm) and an ambient field. Plants and dummies are placed below the walking arena consisting of gauze (possible positions of pots are indicated by (x)). The chamber walls are provided by polyethylene foil (here: cooking bag).

In order to study the weeviĺs olfactory orientation behaviour, we built a new type of olfactometer which mimicked an odorous background around the host plant comparable to the field situation (Fig. 1). This static two-circle olfactometer consisted of circular polyamide gauze (mesh width 0.12 mm, ø 180 mm) that served as a walking arena for the weevils and was divided into a central (ø 60 mm) and an ambient circle. The walking arena was stabilised by metal stands of 40 cm height. Test plants were placed below the walking arena either into the central chamber or into the ambient chamber. The wall of the chambers consisted of flexible polyethylene bags (Toppits ®, Cofresco Frischhalteprodukte GmbH & Co. KG, Minden, Germany) clipped to a glass plate (30 cm×30 cm) at the base of the entire set-up. The ambient chamber provided space for four pots and up to three pots could be placed in the central chamber. A light source (60 W, photosynthetic active radiation 6 µmol m^−2^ s^−1^) was located above the olfactometer in a distance of 50 cm from the walking arena. Prior to each test, the females were allowed to acclimate for one hour in the test room without food. The plants were acclimated in the olfactometer setup for one hour. Bioassays commenced by the release of a single female weevil in the centre of the ambient field at 3 cm distance from the border of the setup. Bioassays were conducted from 10 to 18h under laboratory conditions (22°C±1°C and 43%–65% rh). To avoid diurnal biases, experiments with the same plant arrangement (treatment) were conducted on different days and at different times of day. After every tested female the walking arena was cleaned with ethanol. Odour from one plant arrangement was offered consecutively to five females. The polyethylene foil was changed for every treatment.

In total, N = 20 females were tested separately for one treatment (plants: N = 4). Each female was observed for 300s. In order to evaluate the weeviĺs host plant finding success and search activity, we recorded behavioural parameters by using the software “The Observer 3.0” (Noldus, Wageningen, The Netherlands) (see below for details on behavioural parameters).

### Herbivore olfactory orientation to the host plant in an odorous environment

Table 1 summarizes the different odorous environments in which the weevilś orientation to the host plant was tested. Dummy plants (here referred to as dummies) were built from a pot with soil and a green sheet of paper (210 mm×297 mm) that was rolled up and plugged into the soil. In experiments with vacant zones in the ambient chamber, dummies were placed in the olfactometer to provide consistent visual (colour) stimulation [16]. For control, we tested a dummy in the central and four dummies in the ambient chamber. Attraction to the host plant was tested by placing a flowering *P. lanceolata* in the central chamber and four dummies in the ambient chamber (Table 1, experiment (a)). We used only flowering host plants because these are the target hosts of gravid female weevils. We compared the time the weevil stayed in the central circle (duration of stay) in these experiments.

**Table 1:** Olfactory response of the weevil *Mecinus pascuorum* to host plant odour in the presence of different non-host plant odours in the surroundings.

	Treatment		Duration of stay in central field [s]	Time to reach the central field [s]
	Central field	Ambient field		
(a)	HO	DU	61.6	74.1
			(7.1–124.8)	(30.2–293.0)
(b)	HO + HE	DU	87.9	122.9
			(0.0–155.6)	(80.2–300.0)
(c)	HO	HE + DU	78.0	43.9
			(12.8–139.5)	(17.9–279.6)
(d)	HO	HE + GR	71.2	65.2
			(51.1–112.3)	(27.0–125.5)
		Statistics	n.s.	n.s.

Setup: two-circle olfactometer. Duration of stay in the central field and time to reach the central field (latency) [in seconds] are shown for 20 females tested per treatment observed for 300s. Only dummies (DU) or herbaceous species (HE; *Achillea millefolium* and *Agrimonia eupatoria*) and grass species (GR; *Festuca rubra* and *Poa pratensis*) were presented in different combinations in the ambient, or additional to the host plant, (HO; *Plantago lanceolata*) in the central chamber. Dummies consisted of a pot filled with soil and a green sheet of paper. Medians and interquartile ranges (parentheses) are given. n.s. indicates no significant difference (*P*>0.05) when comparing the different treatments for one behavioural parameter evaluated by Kruskal-Wallis ANOVA followed by Mann-Whitney-*U* test and Bonferroni correction.

Odours from different plants that are placed in the central chamber might be perceived as a single blend by an insect since the odour sources are placed very closely together. In contrast, odour provided by plants in the central chamber and odour released from plants in the ambient chamber might be perceived as separate blends because the odour sources are further apart than those placed altogether in the central chamber. It is well known that successful host location may depend on whether the odour source of a host is detected separately from other odour sources [40]. Therefore, we tested the effect of plant diversity on olfactory host location first by placing non-host plants (two herbaceous species) and the host plant in the central chamber, while four dummies were placed in the ambient chamber (Table 1, experiment (b)). Furthermore, the effect of plant diversity was tested by placing the host plant in the central chamber and two non-host plant species (two herbs) plus two dummies in the ambient chamber (Table 1, experiment (c)). In a further bioassay, we tested how the orientation of the weevil to the odour of the host plant in the central chamber is affected by odour from four different non-host plant species (two herb species and two grass species) placed in the ambient chamber (i.e. higher plant complexity than in the abovementioned bioassay with respect to the number of plant species and amount of biomass) (Table 1, experiment (d)). In all three set-ups a flowering *P. lanceolata* plant was positioned in the central field.

In order to evaluate the weevil’s host plant finding success and search activity, we recorded the following behavioural parameters: time the weevil spent in the central field (duration of stay), time the weevil needed to enter the central field (target odour) for the first time (latency), number of switches between central and ambient field (frequency), total time walking of a weevil during the observation time (activity). The “latency” was defined as the time the weevil needed to enter the central field for the first time during the observation period of 300s; it was set 300s when the weevil had not reached the central field at all. “Frequency” describes how often a field was visited and serves as an indicator of switches between odour fields. Walking activity was measured as the time during which the weevils were actively walking around rather than resting or cleaning themselves.

### Effect of plant diversity on olfactory orientation of the parasitoid

**Insects and plants.** Parasitoids were obtained from *P. lanceolata* inflorescences collected in the Biodiversity Exploratories in July and August 2010. The inflorescences were kept under the same conditions as the ones obtained for the fieldwork data in 2008 (described below). Emerging unparasitised weevils as well as parasitoids emerging from inflorescences infested with weevil larvae were taken out of the boxes every two days. Thereafter the parasitoids were kept at 10°C ± 1°C, 65% rh, 18:6 LD and fed with aqueous honey solution. They were kept at a long-day-period to mimic European summer time and to retard hibernation. Parasitoids were four to six weeks old when tested. In total, 446 male and female parasitoids emerged. Mating opportunities were given after emergence in inflorescence boxes as well as by keeping both sexes together for at least two weeks. Female parasitoids were tested. Because of the high number of replications and a shortage of parasitoids, we had to test each parasitoid about 1.3 times. Parasitoids were pooled after testing. Female parasitoids were chosen randomly from this pool for the next test. Parasitoids could rest at least two days between two consecutive tests. Bioassays were performed in August and September 2010.

All plants that were used for the laboratory assays with parasitoids were grown under the same conditions and used at the same age and phenotypic stage as described above for the plants used for bioassays with weevils. However, seeds of plants used for the bioassay with parasitoids were obtained from Appels Wilde Samen GmbH (Darmstadt, Germany).

### General olfactometer setup

In order to study the parasitoids olfactory orientation behaviour, we used a dynamic Y-tube olfactometer. We tested (i) whether the parasitoid is attracted by odours from the host complex and (ii) how olfactory orientation of the parasitoid is affected by the presence of odour from non-host plants. The two-circle olfactometer used for testing the weeviĺs orientation ability could not be adopted for the parasitoid, since the parasitoid individuals did not adapt to the walking arena and showed only frantic activity with erratic movements.

The Y-tube olfactometer used for testing the parasitoids olfactory behaviour consisted of a Y-shaped glass tube (one 20 cm arm and two 14 cm branched arms, ø: 1.2 cm). The open ends of the branched arms were connected by Teflon tubing to glass jars (2100 ml) containing the odour sources (host complex, plants). Air that entered the glass jars was charcoal-filtered and humidified. Air was pumped with a flow of 138 ml/min through the setup. The flow was controlled by flowmeters (Supelco, Bellefonte, PA, USA). Both the tested odour sources and the parasitoids were acclimatised in the test room one hour before testing. One parasitoid was placed into the opening of the long arm of the Y-tube and was observed for a maximum of 300s. We recorded the number of parasitoids which entered one arm and crossed an imaginary border of 5 cm within this arm. Ten parasitoids were tested for each odour source. After testing ten parasitoids, tubes and glass jars were cleaned with 96% ethanol, heated at 100°C for one hour, and odour source sides were exchanged. Prior to the bioassays with plant odours, a blank test was conducted, and the parasitoids showed no side preference.

### Parasitoid olfactory orientation to the host and host plants in an odorous environment

The host complex consisted of flowering *P. lanceolata* and five female and five male weevil adults. Although the parasitoid attacks only larval host stages, we did not conduct experiments with host plants infested by weevil larvae as weevils kept in the laboratory did not lay eggs; hence, no host plants with larvae-infested seeds were available in the laboratory. We did not collect host plants infested by weevil larvae from the field since it would have been impossible to determine precisely for how long the plants had been infested and thus, no consistent conditions would have been provided when using these plants. However, we took an alternative approach since in preliminary tests the odour of the host complex consisting of adult weevils plus the host plant was attractive for the parasitoid, whereas odour of the host plant and odour of host adults tested separately were not attractive (data not shown). It is well known that parasitoids parasitising inconspicuous hosts or host stages (here: larvae hidden within seeds) may also use cues from non-appropriate host stages (here: host adults) (infochemical detour; [34], [41]). In our study system, weevil females lay their eggs in the seeds of *P. lanceolata* inflorescences in June and July and stay in the habitat where they have oviposited (pers. observation: N. Wäschke); hence, the host plant plus adult host weevils provide a suitable odour source to test the olfactory orientation abilities of the parasitoid.

In order to test the effect of non-host plants on host location, the parasitoids olfactory response to the following combinations was tested: (a) odour of the host complex: flowering host plant *P. lanceolata* with five female and five male weevil adults *versus* a control (N = 50); (b) odour of two non-host plants (*A. millefolium* and *A. eupatoria*) plus host complex *versus* a control (N = 50); and (c) odour of the host complex (see (a)) *versus* odour of the two non-host plants plus host complex (see (b)) (N = 50). As a control we used a pot filled with soil.

### Field study

**Effect of plant diversity on herbivore and parasitoid abundance.** The field study was conducted as a subproject of a German priority project entitled “Biodiversity Exploratories” (described in detail by [35]). In three geographical regions (exploratories) in Germany (from north to south: Schorfheide-Chorin Biosphere Reserve, Hainich-Dün National Park, and Schwäbische Alb Biosphere Area) 50 grassland plots were assigned to biodiversity research. A plot (50 m×50 m) is almost homogenous with respect to soil type and vegetation properties. The three regions across Germany differ in environmental variables, i.e. in precipitation, altitude, and annual mean temperature [35]. The grassland sites are subjected to different land use and thus show differences in plant diversity. Since land use intensity and plant diversity are negatively correlated with each other [42], we neglected land use intensity and focused on plant diversity effects on insect abundance.

The occurrence of the host plant (*P. lanceolata*) in the three regions determined the number of plots studied per region: N = 21 plots in Schorfheide-Chorin, N = 22 in Hainich-Dün, and N = 34 in Schwäbische Alb. Within each plot we sampled ten randomly chosen focal *P. lanceolata* plants distributed across the plot. The number of herbaceous plant species and the vertical coverage of each plant species (r = 15 cm) as well as host plant density (r = 100 cm) around the chosen focal plant were surveyed once in June 2008. To determine the abundance of weevils and parasitoids we collected *P. lanceolata* inflorescences from July to August 2008. Since just a small number of insects might hatch from the ten focal plant inflorescences collected at each plot, we collected additionally 100 randomly chosen *P. lanceolata* inflorescences across the plot by following a random step pattern. Inflorescences of *P. lanceolata* were kept in plastic boxes (17.0 cm×12.5 cm×5.6 cm) with a top cover made of fine-meshed gauze (0.12 mm) under constant conditions (22°C ± 1°C, 50% rh, 11:13 LD). Adult weevils and parasitoids that emerged from the inflorescences in August and September 2008 were identified and counted. In addition to the weevil *M. pascuorum,* adults of a further weevil species emerged, *Mecinus labilis.* We also recorded the number of individuals of this species since it also serves as host for the larval parasitoid. Data are stored at the BExIS database of the Biodiversity Exploratories [43, http://www.biodiversity-exploratories.de/intranet/].

### Statistical analysis

**Laboratory assays.** *Effect of plant diversity on olfactory orientation of the herbivore:* All calculations were performed using R [44]. The Wilcoxon one sample-test was used to evaluate the data obtained by recording the weeviĺs response to the host plant odour (Table 1, a) in the two-circle olfactometer. We tested whether the time spent by the weevils in the central field differed significantly from the null hypothesis (33.3s, assuming equally long residence times in all areas of the olfactometer during an observation period of 300s). If the weevilś duration of stay in the central field differed significantly from 33.3s, the weevils discriminated between odour in the central and the ambient field.

Furthermore, we compared the weevilś olfactory responses to the odours provided by the different odour source combinations by a Kruskal-Wallis ANOVA followed by Mann-Whitney-*U*-tests with Bonferroni correction to account for non-normality of the data [45]. Variance homogeneity was checked by the Levene-test, and if necessary, logarithmic transformation was conducted.

**Effect of plant diversity on olfactory orientation of the parasitoid**. Data obtained from the parasitoid bioassay in the Y-tube olfactometer were analysed by the sign test [46]. Only parasitoids that made a decision were included in the analysis.

### Field study

**Effect of plant diversity on herbivore and parasitoid abundance.** Field data were analysed using a generalised linear mixed model. Plant diversity was calculated according to the Shannon-Index H = – ∑p_i_ × ln p_i_ where p_i_ is the ratio of the i^th^ species compared to the entire pool. We calculated mean values per plot for host plant density and plant diversity. The region was used as a random effect. Explanatory variables with non-normal distribution were ln transformed for stabilising variance [47]. A term was added (+1) before transformation if necessary. Models were calculated by the lmer function with Laplace approximation in R (package lme4 Version 0.999375-37) with a Poisson error distribution (link  =  log) for the abundance data as response variables. To account for overdispersion we added an individual based random effect [48]. We started with the full model and discarded terms that were not significantly different from zero. Models were compared by Akaike Information Criterion (AIC, [49]) until we ended up with a minimal adequate model with the AIC not decreasing anymore or all terms included in the model being significantly different from zero. As fixed effects we added plant diversity and host plant density since the availability of the host plant also impacts upon insect performance and affects the occurrence and abundance of herbivores [6] and their parasitoids [50]. To analyse the effect of vegetation parameters on the parasitoidś abundance we corrected for the host abundance by including this variable in the model as a covariate. For calculating host abundance, adults of both weevil species, *M. pascuorum* and *M. labilis,* were counted.

## Results

### Laboratory assays

**Effect of plant diversity on olfactory orientation of the herbivore.** The two-circle olfactometer proved to be a suitable laboratory device for testing the orientation of the weevils in complex odorous environments. The weevils did not stay significantly longer in the central field compared to the value expected by the null hypothesis (33.3s; assuming equal duration of stay in the entire arena) when dummies were offered in the central and the ambient chamber (median duration of stay in central field: 0s; interquartile ranges: 0–47.2 s; N = 20, *P*>0.05). Weevils stayed significantly longer in the central field with the host plant odour than expected (median duration of stay: 61.6s; interquartile ranges: 7.1–124.8 s; N = 20, *P*<0.05; Table 1, a) and thus were attracted and/or arrested by odours from the flowering host plant *P. lanceolata*.

When comparing the weeviĺs response to odour of the different plant combinations, the duration of stay in the central field and latency of females to reach the central field did not differ between the treatments with the various plant arrangements tested here (χ^2^ = 0.432, χ^2^ = 2.638 respectively, both: *df* = 3, *P*>0.05; Table 1). Neither odour from non-host plants placed additionally to *P. lanceolata* beneath the central field (Table 1, b) nor that of non-host plants offered in the ambient field (Table 1, c) hampered the host finding process. Even though four non-host plants were present in the ambient chamber (Table 1, d), the herbivores still preferred the host plant odour. This indicates that non-host plant odours did not disturb olfactory orientation of female weevils towards flowering host plants.

**Figure 2:**
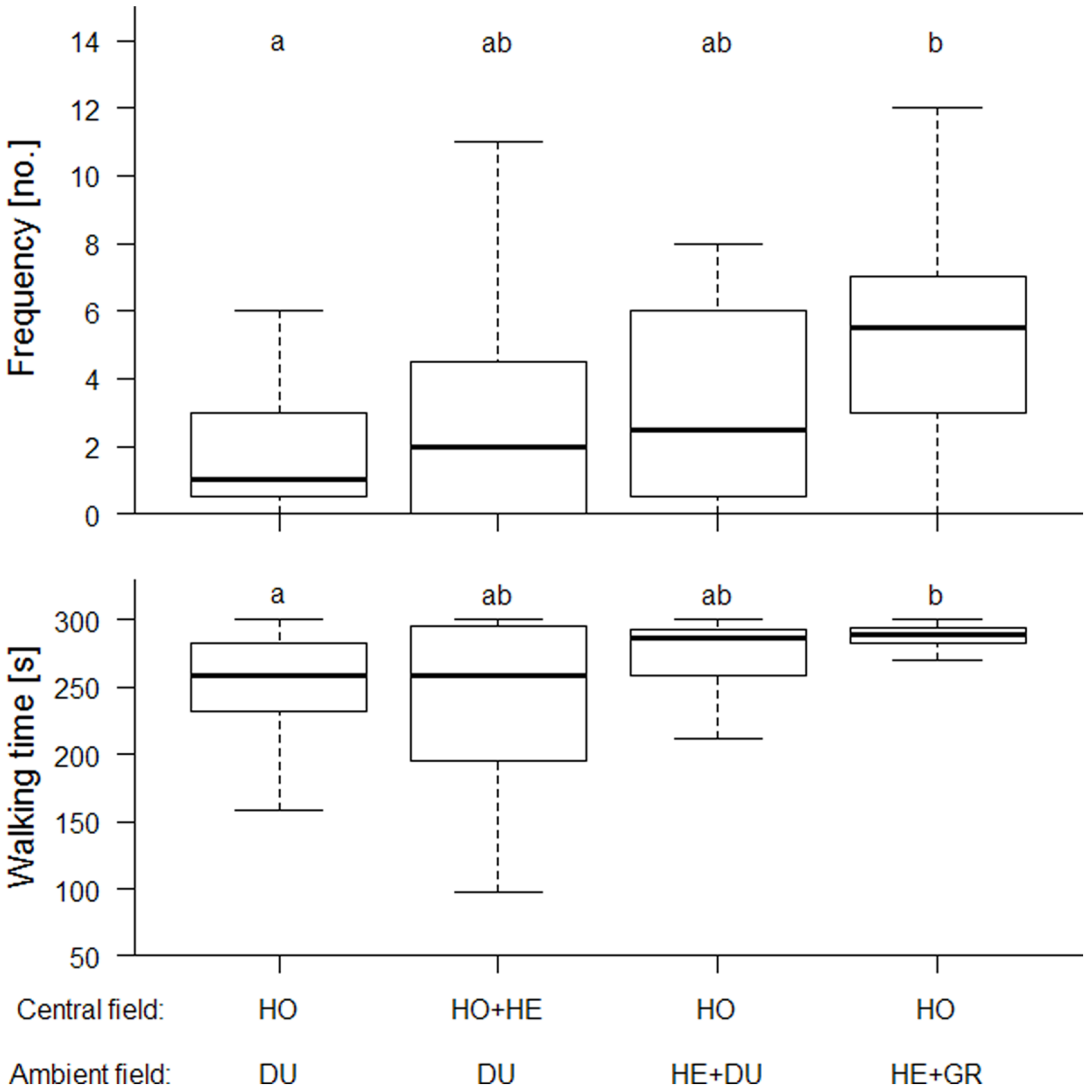
Host plant location of the weevil *Mecinus pascuorum* in different odorous surroundings. The olfactory orientation of female weevils to their host plant *Plantago lanceolata* was tested in the two-circle olfactometer during 300s (N = 20 females per treatment). Number of switches between the fields (frequency) and walking time (in seconds) are shown as medians and quartiles for combinations of host plant (HO) and non-host plant species (HE: herbs; GR: grasses) as well as dummies (DU). Different letters indicate significant (*P ≤* 0.05) differences (Kruskal-Wallis ANOVA followed by Mann-Whitney-*U* test and Bonferroni correction).

However, when comparing the different treatments we observed significant differences in the overall walking activity (χ^2^ = 9.805, *df* = 3, *P*<0.05) and the frequency by which the weevils crossed field borders (χ^2^ = 10.366, *df* = 3, *P*<0.05). These behavioural parameters were enhanced when the weevils experienced odours from the four non-host plants in the ambient chamber additional to the host plant in the central chamber compared to the setup with only *P. lanceolata* placed in the central chamber and no other plants in the ambient chamber (Fig. 2).

**Figure 3:**
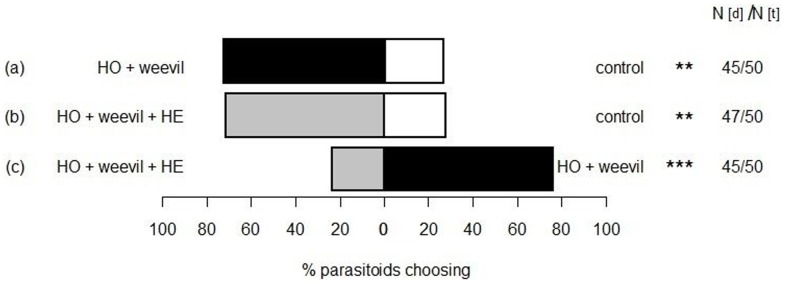
Response of the parasitoid *Mesopolobus incultus* to “host complex” odours in the presence of non-host plants. The % of total number of female parasitoids is shown that responded to odours offered in a Y-tube olfactometer. A pot with soil served as control (white bars). Two different odour sources were used: the host complex (HO: host plant *Plantago lanceolata*, weevils: five male and five female *Mecinus pascuorum*) (black bars) and the host complex plus herbs (HE: *Achillea millefolium* + *Agrimonia eupatoria*) (grey bars). Only parasitoids making a decision were included in the analysis. Numbers of parasitoids making a decision (N_d_) and numbers of tested parasitoids (N_t_) are given. Data were analysed by the sign test according to MacKinnon: **, *P* ≤ 0.01; ***, *P* ≤ 0.001.

**Effect of plant diversity on olfactory orientation of the parasitoid.** Dynamic Y-tube olfactometer studies were conducted to investigate the influence of non-host odours on the orientation of the parasitoid. The parasitoid was attracted to odour from the host complex consisting of flowering *P. lanceolata* and female and male weevils when tested against a control (a pot with soil) (Fig. 3, a). When odour from non-host plants was added to the host complex odour (i.e. increased plant diversity) and tested against a control, this additional non-host plant odour did not affect the attractiveness of the host complex (Fig. 3, b). However, when offering the parasitoids a choice between odour from the host complex only (without non-host plant odour) and odour from the host complex with non-host plant odour added, the parasitoid preferred the simpler odour bouquet (Fig. 3, c). The results indicate that the parasitoid can distinguish between non-host plant and host plant odours.

### Field study

**Effect of plant diversity on herbivore and parasitoid abundance.** To investigate whether abundance of the herbivore and its parasitoid is linked with plant species diversity, we recorded vegetation data in three different regions in Germany (Table 2). The field data revealed a positive correlation of plant diversity with the abundance of the weevils; host plant density had no significant effect on weevil abundance (Table 3). The abundance of the parasitoid was independent of plant diversity, and best explained by the abundance of its hosts (both weevil species: *M. pascuorum* and *M. labilis*). Abundances of the parasitoids and the weevils correlated positively (Table 3).

**Table 2:** Field data: effect of plant diversity on insect abundance.

Region		Host plant density (average per plot) ^a^	Plant diversity (H) (average per plot) ^a^	Abundance of *Mecinus pascuorum* (per plot) ^b^	Abundance of *Mesopolobus incultus* (per plot) ^b^
Schorfheide-Chorin	Central value	14.5±3.1	0.9±0.1	8 (0 – 18)	8 (0 – 29)
(north)	N	21	21	21	21
	Range	1.0 – 47.0	0.0 – 1.4	0 – 32	0 – 161
					
Hainich-Dün	Central value	17.4±3.3	1.5±0.1	1 (0 – 19)	8 (0 – 25)
(central)	N	22	22	22	22
	Range	1.0 – 52.0	0.9 – 2.0	0 – 137	0 – 618
					
Schwäbische Alb	Central value	22.9±4.5	1.7±0.1	0 (0 – 0)	8 (0 – 5)
(south)	N	34	34	34	34
	Range	2.0 – 111.0	0.9 – 2.4	0 – 4	0 – 31

Plant abundance and diversity (H  =  Shannon index), herbivore (*Mecinus pascuorum)* and parasitoid (*Mesopolobus incultus*) abundance are shown recorded in the three regions in Germany. Central tendencies (^a^ mean (SE), ^b^ median (interquartile ranges)), number of plots (N), and ranges for the explanatory and the response variables are given for each region.

**Table 3:** Statistics: effect of plant diversity on insect abundance.

	Abundance of *Mecinus pascuorum*				Abundance of *Mesopolobus incultus*			
Explanatory variables	B	SE	*z* value	*P*	β	SE	*z* value	*P*
Intercept	–5.6026	2.1219	–2.640	**<0.01**	–0.5121	0.2782	–1.841	<0.1
Plant diversity (H)	2.5085	1.0397	2.413	**<0.05 (+)**	NA	NA	NA	NA
Host plant density ^a^	0.5961	0.3311	1.800	<0.1	NA	NA	NA	NA
Host (weevil) abundance ^a^	–	–	–	–	1.1133	0.1216	9.159	**<0.001 (+)**
								
AIC full model	214.7				250.7			
AIC minimal model	214.7				247.5			

Results of a generalised linear mixed model describing the abundances of the herbivorous weevil *Mecinus pascuorum* and the parasitoid *Mesopolobus incultus* in the field. Estimates (β) with standard errors (SE) are given for the minimal adequate model (evaluated by Akaike information criterion (AIC)). *P* values are marked bold if significant. Direction of relationship is given in parenthesis. Seventy-seven plots were involved in the analysis. ^a^ ln transformed; NA: excluded from the model; –: not included in the full model.

## Discussion

We examined the effect of plant diversity on host location behaviour and olfactory orientation of the weevil *M. pascuorum,* a specialist on *P. lanceolata,* and of the pteromalid wasp *M. incultus,* a larval parasitoid of this herbivore. We could demonstrate that (1) female weevils were attracted by odour of flowering *P. lanceolata* plants even when non-host plant odour was present in the surroundings. (2) Similarly, the parasitoid was attracted by odour of its host complex (volatiles from host plant and host) even when this was combined with non-host odour in a no-choice situation. However, the parasitic wasps preferred the “pure” host complex odour when having the choice between the “pure” odour and its combination with non-host plant odour. (3) In the field, increased plant diversity was positively correlated with the herbivorés abundance, but not with the parasitoids abundance; the latter was positively linked with the herbivorés abundance.

### Laboratory assays

In the laboratory we tested the effect of non-host plant odour on the olfactory orientation of the weevil and its parasitoid. A new olfactometer setup was designed, suitable for testing behavioural responses of the weevils to spatially separated odours. It mimics the natural situation where an insect is approaching an odour source surrounded by background odours.

### Effect of plant diversity on olfactory orientation of the herbivore

The herbivorous weevil was attracted by volatiles of flowering *P. lanceolata*. Our study shows that odours of various non-host plants in the presence of host plants did not reduce the weeviĺs success in finding the host plant by olfactory cues. Even when placing non-host and host plants very closely together in the central chamber, the weevils were able to olfactorily detect their host plant within the mixed blend and stayed longer in the central field.

Host location in insects may not only be affected by odours of non-host plants, but also by visual interference with non-host plant neighbours [16], [51]. The walking arena of the olfactometer was built of fine-meshed gauze which made it difficult for the weevils to recognize structures of the plants placed several centimetres below the arena. To minimize the effect of colour, dummies made of green paper were used to simulate the green colour of the plants in the plant-free control field. Hence, structural plant cues were almost undetectable and colour cues were almost the same in all olfactometer fields. Thus, we concluded that orientation of the weevils towards a field was due to olfactory orientation rather than to visual orientation.

Although *M. pascuorum* was attracted to the host plant odour independently of non-host plants in the surroundings, high plant diversity with four non-host plants in the ambient chamber induced increased activity in the females. It has been suggested that intensive motion activity may help insects to separate different odour sources [52]; hence, the high locomotion activity of the weevil *M. pascuorum* in the presence of high plant diversity might support host plant location. In line with these thoughts, one might consider a suitable foraging habitat as an environment that elicits intensified host searching behaviour [53], i.e. increased locomotion activity. Since *P. lanceolata* emits only few volatiles [54], background odour released from co-occurring non-host plants might indicate the presence of a suitable habitat.

The effects of non-host plant odour on host foraging in insects vary with the plant – insect system considered. Non-host odour has been shown to impede host foraging in many insect species (e.g. [55]). In contrast, non-host odours or ubiquitous green leaf volatiles were found to have positive effects on host location of other insects (e.g. [28], [56], [57]). So far, it is not possible to detect common patterns that would allow predicting the impact of non-host odour on insect host foraging success. In our study, a rich odorous environment may stimulate weevils to search more intensively and thus, may improve the likelihood of locating a host plant. Although we cannot distinguish whether the higher weevil activity in the olfactometer was indeed caused by a higher number of plant species or by higher amount of plant biomass and thus a higher amount of volatiles, our laboratory and field data suggest that odour of vegetation with greater plant diversity presents a “patch of interest” for the weevil. Patches with host plants and high non-host plant diversity in the surroundings may provide enhanced host plant quality or offer more refuge areas allowing weevils to escape from natural enemies or competitors [58].

### Effect of plant diversity on olfactory orientation of the parasitoid

The larval parasitoid was attracted by the host complex odour (i.e. odour from the host plant and the host insect, i.e. the weevils) and was capable of discrimination between host complex odour offered with and without non-host plant odour. The parasitoid responded more strongly to the simpler odour bouquet lacking non-host odour, but was also attracted to the odour of the host complex offered together with non-host plants. Parasitoids do not only use volatile cues from the host or the host plant for host location, but also cues emitted by the host habitat [59]. In the tritrophic system studied here the weevils lay their eggs in June and July and remain in the habitat where they have oviposited. The emission of volatiles from *P. lanceolata* was found to significantly increase after herbivory by generalist larvae that severely damaged the plant [54]. However, adult *Mecinus* weevils chew only upon small parts of the flower stem. Therefore, the adult weevils are not expected to induce *P. lanceolata* in such a way that it leads to a significantly higher volatile emission. The quantity of emitted and perceived plant volatiles is important for parasitoids when searching for herbivorous hosts [25]. Thus, it might be beneficial for the parasitoid to positively respond to general cues from the habitat where the host with its host plant occurs. In conclusion, the parasitoid may use habitat odour for long-range orientation and might be attracted to the host complex in combination with non-host plants as habitat odour. As shown for different parasitoid species, the presence of non-host plants does not always hinder the close-range foraging activities [60]. However, the parasitoid may respond specifically to the pure host complex at a short-range scale when a choice between odours of the host complex and the surroundings is possible. This was shown here where the parasitoid distinguishes between the host complex offered alone and in combination with non-host plants.

### Field study

**Effect of plant diversity on herbivore and parasitoid abundance.** In agricultural habitats the abundance of specialised herbivorous insects was shown to decrease with increasing plant species diversity, thus indicating that specialist herbivores are negatively affected by plant diversity (reviewed by [16], [61]). In contrast, when considering plant - herbivore interactions of non-crop species in a natural or semi-natural context, beside negative effects of plant diversity [14], several studies found a positive effect of high plant diversity on plant damage by herbivory [62], on the probability of herbivore occurrence [12], and on herbivore abundances [7]. The results of our study corroborate these latter findings.

In our field study the abundance of the parasitoid of weevil larvae was strongly associated with host abundance. The positive correlation found between abundances of weevils and parasitoids might be explained by improved oviposition possibilities for the parasitoids in patches with high host density, thus leading to an aggregation of parasitoids in said patches [63]. The abundance of the herbivorous host considered here is positively correlated with plant diversity which often correlates positively with herbivore diversity [64]. Since the studied parasitoid species can parasitize also other insect hosts in the same habitat, not only higher host density but also higher host diversity could be an explanation for the positive correlation between herbivore and parasitoid abundance in this study [65]. However, parasitoid abundances are often not affected by just a single environmental factor [11], [64].

## Conclusions

In our laboratory bioassays we have shown that both an herbivorous and parasitic insect are not prevented from successful host location when plant diversity increases. Furthermore, the laboratory study revealed that odour from a species-rich vegetation enhanced the weevilś searching activity for host plants. In the field, the abundance of herbivorous weevils was positively correlated with plant diversity. In grasslands, diverse habitats may constitute high quality patches where numerous multitrophic interactions characterise complex food webs. To gain a deeper understanding of the mechanisms which shape the positive relationship between communities of high plant species diversity and the organisms involved in multitrophic interactions, further laboratory and field studies are necessary. These should experimentally alter the chemical diversity of habitat odour and disentangle odour-mediated plant diversity effects on insect abundances from other parameters that vary with changing plant species diversity.
